# Phase I clinical trial designs in oncology: A systematic literature review from 2020 to 2022

**DOI:** 10.1017/cts.2024.599

**Published:** 2024-09-24

**Authors:** Ning Li, Xitong Zhou, Donglin Yan

**Affiliations:** 1 Department of Biostatistics, College of Public Health, University of Kentucky, Lexington, KY, USA; 2 Markey Cancer Center, University of Kentucky, Lexington, KY, USA; 3 Dr. Bing Zhang Department of Statistics, University of Kentucky, Lexington, KY, USA

**Keywords:** Dose-escalation design, dose expansion, dose-limiting toxicity, maximum tolerated dose, phase i trials

## Abstract

**Background:**

Phase I clinical trials aim to find the highest dose of a novel drug that may be administrated safely without having serious adverse effects. Model-based designs have recently become popular in dose-finding procedures. Our objective is to provide an overview of phase I clinical trials in oncology.

**Methods:**

A retrospective analysis of phase I clinical trials in oncology was performed by using the PubMed database between January 1, 2020, and December 31, 2022. We extracted all papers with the inclusion of trials in oncology and kept only those in which dose escalation or/ and dose expansion were conducted. We also compared the study parameters, design parameters, and patient parameters between industry-sponsored studies and academia-sponsored research.

**Result:**

Among the 1450 papers retrieved, 256 trials described phase I clinical trials in oncology. Overall, 71.1% of trials were done with a single study cohort, 56.64% of trials collected a group of at least 20 study volunteers, 55.1% were sponsored by industry, and 99.2% of trials had less than 10 patients who experienced DLTs.

The traditional 3 + 3 (73.85%) was still the most prevailing method for the dose-escalation approach. More than 50% of the trials did not reach MTDs. Industry-sponsored study enrolled more patients in dose-escalation trials with benefits of continental cooperation. Compared to previous findings, the usage of model-based design increased to about 10%, and the percentage of traditional 3 + 3 design decreased to 74%.

**Conclusions:**

Phase I traditional 3 + 3 designs perform well, but there is still room for development in novel model-based dose-escalation designs in clinical practice.

## Introduction

Phase I clinical trials conducted in oncology aim at identifying the appropriate dosage for further examination, commonly referred to as the recommended phase II dose (RP2D). Trials are designed to gather data regarding the drug’s safety, pharmacokinetics, and mechanism of action. The primary objective of dose-finding studies is to find a novel drug’s maximum tolerated dose (MTD) that can be administered without causing excessive toxicity. Dose-limiting toxicities (DLT) are defined as severe adverse events (AE) of grade 3 or higher which were usually prespecified according to the Common Terminology Criteria for Adverse Events (CTCAE v4.0) [1]. The CTCAE is a set of global standards that gauge the seriousness of an AE, spanning from a mild level (grade 1) to events linked to death (grade 5) [2]. Traditionally, phase I dose-finding designs are typically categorized into algorithm-based and model-based approaches [3,4]. Algorithm-based designs perform predetermined guidelines to conduct dose-escalation and de-escalation procedures, which include the traditional 3 + 3 design, accelerated titration design [5], the biased-coin design [6], and its variations [7,8] . The traditional 3 + 3 design is the most widely used algorithm-based design in clinical practice. The design is easy to implement. It begins at a low dose and escalates with every 3–6 patients for each dose. Initially, three patients were enrolled, and additional patients were enrolled into higher dose level if no DLT occurred [4,9]. If a particular dose leads to the occurrence of a DLT in one patient, three more patients are added to the same dose-level cohort. The MTD is exceeded if two or more patients out of the cohort experience DLTs at a particular dose level. Then dose escalation is terminated in the trial. Lower dose level needs to expand to six patients to check the DLTs; if no more than one patient has DLT, that dose level is defined as the MTD [10]. But these designs usually have inadequate operational qualities and are without theoretical fundament [3,4]. Model-based designs perform better than algorithm-based designs by using parametric dose toxicity models to determine the dosage for escalating or de-escalating procedures. The continual reassessment method, the most classic model-based design in phase I clinical trials, starts with a probable dose-toxicity curve based on statistical models with one or two parameters and then uses the cumulative toxicity data from trial participants to update the estimation of the curve on a regular basis, which helps determine the appropriate dose [11]. Numerous continual reassessment method (CRM) extensions have been developed, including dose escalation with overdose control [12], time-to-event CRM [13], Bayesian model averaging CRM [14], partial order CRM [15] and its variations [16], and bivariate CRM [17]. For a comprehensive review of the CRM and related methods can be found in the book by Cheung [18]. There are several reasons to prefer model-based designs to algorithm-based designs: clearer DLT, more patients treated at the optimal dose, more efficient utilization of available resources, and easier alternating process to fit complex questions [19]. Contemporarily, though model-based dose-escalation design proposes better solutions to find MTD than algorithm-based ones, their utilization in clinical practice has been limited [4,20,21]. However, because model-based designs require repeated model fitting and estimation, they are more complicated to implement, and many practitioners view the decisions of the model-based designs as coming from a “Blackbox” [22]. Model-assisted designs merged the benefits of algorithm-based designs and model-based design [23–25]. The model-assisted designs employed a certain statistical model to create decision-making frameworks and determines the dose-escalation and de-escalation approach prior to trial, so it allows for straightforward implementation like algorithm-based designs [23]. Model-assisted designs include the Bayesian optimal interval (BOIN) [26,27], the modified toxicity probability interval (mTPI) design and its variation [28],mTPI-2 [28], and keyboard design [25]. Comparison between model-assisted designs with the 3 + 3 design and model-based designs indicated that model-assisted designs significantly outperformed the 3 + 3 design and demonstrated performance compared to model-based designs in accuracy of MTD identification, patient allocation, and the risk of overdosing patients [23,24,29,30] BOIN yielded better outcomes than other model-assisted designs in various trials including drug-combination trials [31], late-onset toxicity [32], low-grade toxicities [32], and toxicity and efficacy jointly [33,34].

Previous review studies found only 1.6% of phase I trials from 1991 to 2006 implemented model-based or assisted designs [20], and 5.4% of trials used model-based designs from 2008 to 2014 [21]. Most recently, a review study of phase I trials reported that model-based designs and model-assisted designs were used in 7% and in only 1%, respectively, of the selected studies from 2014 to 2019. In order to adapt to the needs of cancer research and treatment, it was necessary to update the traditional approaches to phase I clinical trials in oncology [35,36]. However, the incorporated rate of model-based designs in practice has not significantly increased in the past three decades. This literature review aims to assess the trend of the recent phase I clinical trials in oncology over the 2020–2022 period and to evaluate the detailed design characteristics between academic studies and industry-sponsored studies on phase I design types, number of cohorts, study sites, study populations, types of dose escalations, and characteristics of escalation.

## Methods

In this research, we performed a retrospective analysis by compiling a dataset consisting of 1450 studies retrieved from the PubMed database. The selection process was guided by specific search criteria to ensure the relevance and specificity of the chosen studies. This selection process was undertaken to ensure that the studies included in our analysis were relevant to the evaluation of phase I clinical trials in the field of oncology. The chosen studies were subjected to further examination and analysis as part of our research methodology.

### Search strategy

To gather the initial pool of studies, we conducted a search using the PubMed database, employing the following key terms: “phase I” and “clinical trials.” This initial search yielded 1450 results from 2020 to 2022.

### Inclusion and exclusion criteria

**Figure 1.**
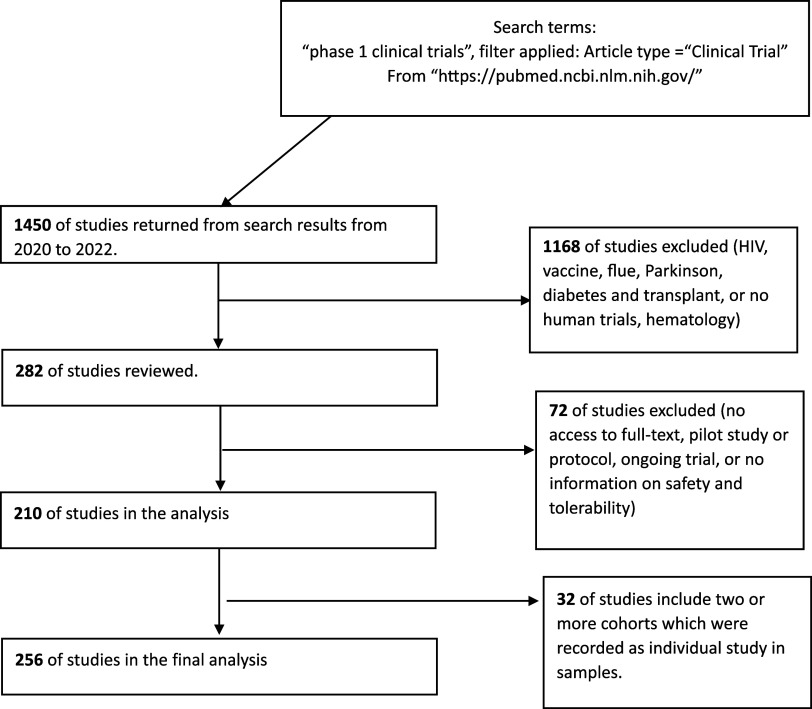
Study selection.

From the initial 1450 studies retrieved, we implemented a systematic exclusion process to narrow down the dataset to studies pertinent to our research focus. First, we excluded 1168 studies that did not pertain to oncology, given the specific nature of our investigation. Second, 72 studies were not included in the analysis due to access restrictions. Consequently, we identified and retained 210 studies that met our inclusion criteria for the final analysis; 32 studies included two or more cohorts recorded as individual studies. Finally, 256 studies reported on dose escalation and/or dose expansion in oncology trials remained for final analysis (Figure 1).

### Data acquisition and validation

The data collection was conducted through the Research Electronic Data Capture system (REDcap). Two graduate students in statistics and biostatistics were trained in common phase I dose-escalation methodologies, inclusion/exclusion criteria, and data collection instruments. They collectively extracted data from 210 articles. 10% of the articles were reviewed by the supervising faculty for training and quality control purposes.

We summarized each study by three groups of parameters: study parameters, design parameters, and patient parameters. Study parameters describe the overall characteristics of the study, including study phase, number of study sites, study sponsor, country, and the number of investigational agents. Study phases are categorized into phase I-a, phase I-b, phase I with expansion, and phase I/II. It is worth noting that the subcategorization of phase I studies lacks formal standardization. For the purpose of this review, we have assigned the label “phase I-a” to studies exclusively featuring dose-escalation protocols, while “phase I-b” designates studies with dose escalation as their sole focus. “Phase I/II” denotes trials that start with a dose-escalation design and subsequently transition to a randomized phase II design. Phase I studies that start with a dose-escalation design followed by a single cohort dose expansion have their own category labeled as “phase I with expansion.”

The number of study sites is categorized into “single site” or “multi-site” studies. Study sponsors are grouped into academic research or industry sponsors. Study sponsors are distinguished by the attached study disclosure. Studies that are funded by government grants or institutional funds are categorized as academic research. When it is unclear in the study disclosure, we examined whether the corresponding and first author is affiliated with an academic institution or a pharmaceutical company. The other study parameters are extracted from each study’s introduction section.

Design parameters include the total number of patients enrolled, the dose-escalation method and its parameters, dose-expansion parameters, and safety statistics. Dose-finding methods include 3 + 3 design, titration design, CRM and modified CRM design, time-to-event CRM, and BOIN. Studies that did not adopt the abovementioned designs are categorized into “others” due to small numbers, and no method information provided. This “other” category includes rolling six design, mTPI, 4 + 3 design, 4 + 4 design, 5 + 2 dose intensification, modified Fibonacci design, and designs without information. For studies with dose-escalation part, we extracted key design elements including the number of candidate dose, starting dose, final dose, dosing finding objective (MTD vs. optimal biological dose [OBD]), and whether the dose escalation involves multiple agents. In this research, the final dose was defined as MTD or the highest dose level if MTD was not reached or OBD.

The total number of patients enrolled in our analysis represents the cumulative sample size across all the studies examined. It’s important to note that some studies employ a design in which patients are enrolled into parallel cohorts. Essentially, these parallel cohorts are multiple groups of patients within the same study, with each group receiving a similar treatment scheme. Although these parallel cohorts are included in a single study report, we treat them as distinct studies during our analysis. It is worth noting that a small number of dose-escalation studies only reported the number of patients treated at each dose level without details on their dose-escalation methods. These studies are categorized as “not available (N/A).” For studies with dose expansion, we extracted the number of patients enrolled for dose expansion.

Patient safety is summarized by the percentage of patients treated at the final dose level, percentage of patients who experienced DLT overall, and percentage of patients who experienced DLT at the final dose level.

We also summarized the characteristics of these three parameters between industry and academia-sponsored trials. Comparisons between industry and academia-sponsored trials were conducted using Chi-square tests and Fisher’s tests if the sample size was less than 5. Chi-square tests and Fisher’s tests were also used to compare the difference between dose-escalation methods. The Haldane-Anscombe correction was applied to fix the comparison for data cells with zero observations by adding 0.5 to each data cell [37,38].

## Results

The 256 phase I trials included 144 trials that only conducted dose escalation, 38 trials that underwent single cohort expansion without dose escalation, 47 trials combined dose escalation and expansion, and 27 dose-escalation trials followed by randomized phase II. About 55% (141 out of 256) of the trials were sponsored by industry. Ninety-nine percent of trials had at most 10 patients with (or experiencing) DLTs. The traditional 3 + 3 (73.85%) remains the most widely used approach for phase I cancer dose-finding clinical trials (Table 1).

**Table 1.** Overall phase I clinical trials in oncology characteristics and comparison between industry and academia sponsored from 2020 to 2022

	Total number *N* = 256	Industry sponsored *N* = 141	Academic research *N* = 115
**Phase 1 type, *n* (%)**			
Phase 1 (first in human or only had dose escalation)	144 (56.25%)	75 (53.19%)	69 (60%)
Phase 1b (single cohort without dose escalation)	38 (14.84%)	20 (14.18%)	18 (15.65%)
Phase ½ (dose escalation followed by randomized phase 2)	27 (10.55%)	17 (12.06%)	10 (8.7%)
Phase 1 with dose expansion (first in human/escalation followed with single cohort expansion)	47 (18.36%)	29 (20.57%)	18 (15.65%)
**Multiple sites, *n* (%)**			
Yes	160 (62.89%)	102 (72.86%)	58 (50.43%)
No	95 (37.11%)	38 (27.14%)	57 (49.57%)
**Study population, *n* (%)**			
0–10	36 (14.06%)	19 (13.48%)	17 (14.78%)
11–20	75 (29.3%)	32 (22.7%)	43 (37.39%)
20+	145 (56.64%)	90 (63.83%)	55 (47.83%)
**Study country, *n* (%)**			
USA (or USA with another country)	133 (51.95%)	62 (43.97%)	71 (61.74%)
Multiple countries	52 (20.3%)	35 (24.82%)	17 (14.78%)
Japan	28 (10.93%)	24 (17.02%)	4 (3.48%)
Other Asian countries (China, Korea, Singapore, etc.)	18 (7.03%)	5 (3.55%)	13 (11.3%)
European countries (UK, France, Italy, etc.)	19 (7.42%)	13 (9.22%)	6 (5.22%)
Other countries	6 (2.34%)	1 (0.71%)	5 (4.35%)
**Agents, *n* (%)**			
Single agents	137 (53.52%)	76 (53.9%)	61 (53.04%)
2 or 2+	119 (46.48%)	65 (46.1%)	54 (46.96%)
**% of patients experienced DLT, *n* (%)**			
0%–10%	185 (72.55%)	107 (75.89%)	78 (68.42%)
11%–20%	41 (16.08%)	22 (15.6%)	19 (16.67%)
21%–30%	17 (6.67%)	8 (5.67%)	9 (7.89%)
30%+	12 (4.71%)	4 (2.84%)	8 (7.02%)
**Number of patients experience DLT, *n* (%)**			
0–10	253 (99.22%)	140 (99.29%)	113 (99.12%)
11–20	1 (0.39%)	0 (0)	1 (0.88%)
20+	1 (0.39%)	1 (0.71%)	0 (0)

	Dose escalation *N* = 218	Industry sponsored *N* = 121	Academic research *N* = 97
**Dose-escalation method, *n* (%)**			
3 + 3	161 (73.85%)	88 (72.73%)	73 (75.26%)
Titration design	6 (2.75%)	5 (4.13%)	1 (1.03%)
CRM or modified CRM	9 (4.13%)	7 (5.79%)	2 (2.06%)
Time-to-event CRM	5 (2.29%)	3 (2.48%)	2 (2.06%)
BOIN	7 (3.21%)	3 (2.48%)	4 (4.12%)
The rolling six design	6 (2.75%)	3 (2.48%)	3 (3.09%)
mTPI	3 (1.38%)	2 (1.65%)	1(1.03%)
4 + 3	1 (0.46%)	1 (0.83%)	0 (0)
4 + 4	1 (0.46%)	0 (0)	1 (1.03%)
5 + 2 dose intensification	1 (0.46%)	0 (0)	1 (1.03%)
Modified Fibonacci design	1 (0.46%)	0 (0)	1 (1.03%)
NA	17 (7.8%)	9 (7.44%)	8 (8.25%)
**Number of patients for escalation, *n* (%)**			
1–10	28 (12.84%)	16 (13.22%)	12 (12.37%)
11–20	64 (29.36%)	25 (20.66%)	39 (40.21%)
20+	116 (57.8%)	80 (66.11%)	46 (47.43%)
**Number of patients at final dose, *n* (%)**			
1–10	185 (84.87%)	103 (85.83%)	82 (88.17%)
10+	33 (15.13%)	17 (14.17%)	11 (11.83%)
**Dose level (top five), *n* (%)**			
2 levels	56 (25.69%)	32 (26.45%)	24 (24.74%)
3 levels	53 (24.31%)	26 (21.49%)	27 (27.84%)
4 levels	35 (16.06%)	16 (13.22%)	19 (19.59%)
5 levels	22 (10.09%)	15 (12.4%)	7 (7.22%)
6 levels	20 (9.17%)	12 (9.92%)	8 (8.25%)
**Target dose, *n* (%)**			
MTD	213 (97.7%)	118 (98.33%)	95 (100%)
OBD	2 (0.92%)	2 (1.67%)	0 (0)
**Start dose, *n* (%)**			
Lowest	209 (95.87%)	116 (95.87%)	93 (95.88%)
Middle	2 (0.92%)	1 (0.83%)	1 (1.03%)
Highest	7 (3.21%)	4 (3.31%)	3 (3.09%)
**MTD, *n* (%)**			
Above highest	105 (48.39%)	66 (54.55%)	39 (40.63%)
Middle	59 (27.19%)	29 (23.97%)	30 (31.25%)
Highest	29 (13.36%)	16 (13.22%)	13 (13.54%)
Lowest	19 (8.76%)	9 (7.44%)	10 (10.42%)
Below lowest	5 (2.3%)	1 (0.83%)	4 (4.17%)
**Multiple agent escalation, *n* (%)**			
Yes	40 (18.87%)	23 (19.66%)	17 (17.89%)
No	172 (81.13%)	94 (80.34%)	78 (82.11%)

	Dose expansion *N* = 85	Industry sponsored *N* = 49	Academic research *N* = 36
**% of patients had DLT at MTD/OBD, *n* (%)**			
0%–10%	67 (78.82%)	35 (71.43%)	32 (88.89%)
11%–20%	4 (4.71%)	4 (8.16%)	0 (0)
20%+	14 (16.47%)	10 (20.4%)	4 (11.11%)
**Number of patients treated at dose expansion, *n* (%)**			
1–10	21 (24.71%)	9 (18.37%)	12 (33.33%)
11–20	21 (24.71%)	13 (26.53%)	8 (22.22%)
20+	43 (50.58%)	27 (55.1%)	16 (44.45%)

DLT = dose-limiting toxicities; CRM = continual reassessment method; BOIN = Bayesian optimal interval; mTPI = modified toxicity probability interval; MTD = maximum tolerated dose; OBD = optimal biological dose.

### Industry study vs. academia study

Industry studies enrolled more patients than academic study in dose escalation (*p* = 0.03) (Table 2). A total of 63.8% of industry studies recruited more than 20 patients; meanwhile, only 47.8 % of academic studies enrolled more than 20 patients. In dose expansion, the percentage of trials including more than 20 patients was 55.1% for industry studies and 44.45% for academic studies, respectively (Table 1). The number of patients at final dose, dose levels, and multiple agent escalation were spread quite evenly for both types of study. There were no significant differences in study design method (*p* = 0.58) and the percentage of DLT (*p* = 0.34) between industry and academia study (Table 2). Industry-sponsored studies enrolled more patients in dose-escalation trials with the benefits of continental cooperation. Industry-sponsored phase I trials tend to involve multiple sites (72.86%) rather than perform trials in a single site, compared to 50% multiple sites for academia-sponsored research (Table 1).

**Table 2.** Association between key characteristics (study design method, percentage of DLT, multiple sites, and study population) with study sponsorship

	Industry sponsored *N* = 141	Academic research *N* = 115	*P*-value
**Study population, *n* (%)**			0.02
0–10	19 (13.48%)	17 (14.78%)	
11–20	32 (22.7%)	43 (37.39%)	
20+	90 (63.83%)	55 (47.83%)	
**Multiple sites, *n* (%)**			0.002
**Yes**	102 (72.86%)	58 (50.43%)	
**No**	38 (27.14%)	57 (49.57%)	
**Phase 1 type, *n* (%)**			0.58
Phase 1 (first in human or only had dose escalation)	73 (51.77%)	66 (57.39%)	
Phase 1b (single cohort without dose escalation)	20 (14.18%)	19 (16.52%)	
Phase ½ (dose escalation followed by randomized phase 2)	17 (12.06%)	10 (8.7%)	
Phase 1 with dose expansion (first in human/escalation followed with single cohort expansion)	31 (21.99%)	20 (17.39%)	
**% of patients experienced DLT, *n* (%)**			0.34
0%–10%	107 (75.89%)	78 (68.42%)	
11%–20%	22 (15.6%)	19 (16.67%)	
20%+	12 (8.51%)	17 (14.91%)	

DLT = dose-limiting toxicities.

*Chi-square tests for categorical variables, the significance level is 0.05.

### Dose-escalation method

The top 3 most famous methods for dose escalation were traditional 3 + 3 design, CRM, and BOIN. Most papers assumed the dose-toxicity relationship was monotonic and increasing. About 97.7% of selected papers targeted to identify MTD, and 95.9% of papers started at the lowest dose. A total of 48.39% of dose-escalation trials did not reach their MTD throughout the trial, and only 2.3% of trials identified the MTD below the lowest dose (Table 3). By comparing the dose-escalation methods, we noticed that there was a significant difference in DLT rate (*P* < .0001) (Table 4). Compared to the extent finding in the previous literature review on phase I trials in oncology [39], the usage of traditional 3 + 3 designs decreased by about 14% (from 88% to 74%), and more model-based designs and model-assisted designs, such as CRM and BOIN, were used to seek the toxicity profile of new drugs or interventions (Table 3).

**Table 3.** Compare dose-escalation method in phase I clinical trials in oncology from 2020 to 2022

	3 + 3 *N* = 161	CRM *N* = 14	BOIN *N* = 7	Titration *N* = 6	Other^a^ *N* = 30
**Number of patients enrolled for dose escalation, *n* (%)**					
1–10	35 (21.88%)	2 (14.29%)	1 (14.29)	3 (50%)	4 (13.33%)
11–20	59 (36.88%)	4 (28.57%)	0 (0)	2 (33.33%)	13 (43.33%)
21–30	37 (23.13%)	6 (42.86%)	1 (14.29)	1 (16.67%)	6 (20%)
30+	29 (18.13%)	2 (14.29%)	5 (71.43)	0 (0)	7 (23.33%)
**Number of patients enrolled for dose expansion, *n* (%)**					
1–10	17 (30.91%)	1 (20%)	0 (0)	0 (0)	0 (0)
11–20	9 (16.36%)	0 (0)	2 (66.67%)	0 (0)	3 (42.86%)
21–30	9 (16.36%)	2 (40%)	0 (0)	0 (0)	0 (0)
30+	20 (36.36%)	2 (40%)	1 (33.33%)	1 (100%)	4 (57.14%)
**Start dose, *n* (%)**					
Lowest	153 (95.03%)	14 (100%)	7 (100%)	5 (83.33%)	30 (100%)
Middle	2 (1.24%)	0 (0)	0 (0)	0 (0)	0 (0)
Highest	6 (3.73%)	0 (0)	0 (0)	1 (16.67%)	0 (0)
**MTD, *n* (%)**					
Below lowest	4 (2.5%)	0 (0)	1 (7.14%)	0 (0)	0 (0)
Lowest	17 (10.63%)	0 (0)	1 (7.14%)	0 (0)	1 (3.33%)
Middle	45 (28.13%)	4 (57.14%)	5 (35.71%)	1 (16.67%)	4 (13.33%)
Highest	23 (14.38%)	0 (0)	1 (7.14%)	2 (33.33%)	3 (10%)
Above highest	71 (44.38%)	3 (42.86%)	6 (42.86%)	3 (50%)	22 (73.33%)
**Number of patients at final dose, *n* (%)**					
1–10	139 (89.1%)	11 (78.57%)	4 (57.14%)	6 (100%)	25 (83.33%)
11–20	14 (8.97%)	2 (14.29%)	1 (14.29%)	0 (0)	4 (13.33%)
21–30	0 (0)	1 (7.14%)	1 (14.29%)	0 (0)	1 (3.33%)
30+	3 (1.92%)	0 (0)	1 (14.29%)	0 (0)	0 (0)
**% of patients experienced DLT, *n* (%)**					
0%–10%	106 (65.84%)	9 (64.29%)	6 (85.71%)	5 (83.33%)	25 (86.21%)
11%–20%	33 (20.5%)	1 (7.14%)	0 (0)	1 (16.67%)	4 (13.79%)
21%–30%	12 (7.45%)	3 (21.43%)	0 (0)	0 (0)	0 (0)
30%+	10 (6.21%)	1 (7.14%)	1 (14.29%)	0 (0)	0 (0)
**Number of dose levels, *n* (%)**					
Less or equal to 5	122 (75.78%)	8 (57.14%)	6 (85.71%)	6 (100%)	26 (85.67%)
More than 5	39 (24.22%)	6 (42.86%)	1 (14.29%)	0 (0)	4 (13.33%)

CRM = continual reassessment method; BOIN = Bayesian optimal interval; MTD = maximum tolerated dose; DLT = dose-limiting toxicities.

a “other” category includes rolling six design, mTPI, 4 + 3 design, 4 + 4 design, 5 + 2 dose intensification, modified Fibonacci design, and designs without information.

**Table 4.** Association between dose-escalation study design method and percentage of DLT

	3 + 3 *N* = 161	CRM *N* = 14	BOIN *N* = 7	Titration *N* = 6	Other^a^ *N* = 30	*P*-value
**% of patients experienced DLT, *n* (%)**						< 0.0001*
0%–10%	106 (65.84%)	9 (64.29%)	6 (85.71%)	5 (83.33%)	25 (86.21%)	
11%–20%	33 (20.5%)	1 (7.14%)	0 (0)	1 (16.67%)	4 (13.79%)	
21%–30%	12 (7.45%)	3 (21.43%)	0 (0)	0 (0)	0 (0)	
30%+	10 (6.21%)	1 (7.14%)	1 (14.29%)	0 (0)	0 (0)	

DLT = dose-limiting toxicities. CRM = continual reassessment method; BOIN = Bayesian optimal interval.

* Fisher’s exact test for categorical variables, the significance level is 0.05

a “other” category includes rolling six design, mTPI, 4 + 3 design, 4 + 4 design, 5 + 2 dose intensification, modified Fibonacci design, and designs without information.

## Discussion

This systematic literature review aims to summarize the characteristics of phase I clinical trials from 2020 to 2022. The review focused on the oncology trials with single- or multiple-agent targeted therapies for single cohort dose escalation and/or dose expansion. We reported summary statistics of the key design parameters as a reference for future study design. We also highlighted the difference between industry sponsored and academia sponsored in the trial’s sample size, design method, and number of patients experiencing DLTs. Also, we summarized the dose-escalation method types, sample size, number of patients at final dose, and percentage of patients experiencing DLT overall as well as industry sponsored versus academic sponsored. The analysis of subgroups revealed that industry-sponsored trials were more likely to recruit more patients than the trials from academic institutions. There were approximately 64% of industry-sponsored trials with at least 20 patients, but only 48% of trials in academic research. Our data showed a significantly higher percentage of multiple sites involved in industry-sponsored trials, compared to the academic research (*p* = 0.002) (Table 2). The difference between academic and industry-sponsored studies is expected because industry studies typically have more resources and infrastructure to recruit patients than single research centers. We found an increase in the percentage of academic sponsored trials among all registered trials globally; this is an improvement supported by existing literature [40].

Other analyses pointed out the difference in trial sponsorship linked to dose escalation and dose expansion in treated patients and the percentage of patients who had DLT at MTD/OBD, which is consistent with our general findings that industry-sponsored trials usually have more trials with at least 20 patients in dose escalation (66.11%), compared to 47.43% among academic sponsored research. A globalization study to examine the difference between industry- and non-industry-sponsored clinical trials pointed out that industry-sponsored trials may have more capacity to perform extensive trials than academic research [41]. Hence, according to phase I clinical trials, industry-sponsored trials may have more dose levels than of academic research; dose levels would be an important confounder in comparing the number of patients enrolled across different sponsorships. While other features in escalation and expansion among academic and industry-sponsored trials remain constant, further studies should be carried out for better comparison in key features. Comparing the percentage of patients experienced aimed to describe the difference among dose-escalation methods. It might not be appropriate to simply compare the rate because the predetermined rate of DLT for CRM usually ranged from 20% to 30% and the rate of DLT to define MTD for the 3 + 3 method was usually one patient out of six patients [42].

The traditional 3 + 3 method in phase I clinical trials in oncology still dominated and functioned well in the real world. However, incorporating newer techniques, methodologies, and patient-centered approaches or increasing the number of patients in the trials has the potential to improve traditional algorithm-based designs [10]. Even though model-based designs developed late, the approach has already had a breakthrough theoretically. The implementation of model-based designs has several barriers that include time constraints, lack of statistical resources, limited practical models or examples from previous publications, and few funding [43].

In conclusion, phase I clinical trial designs in oncology should be promoted, and model-based designs should be implemented widely in the dose-escalation approach. Our analysis did not include trials for which we only had access to abstracts but not to full texts, which might introduce selection bias. The period for the phase I clinical trials publication date was only from 2020 to 2022, which may be too short to reveal trends. It also overlapped the COVID-19 global pandemic when the majority of phase I clinical trials were dedicated to developing the anti-coronavirus treatment or vaccines, resulting in the paucity of phase I clinical trials for oncology compare to the last two decades. Thus, this study should encourage further analysis of the complete picture of the phase I clinical trials’ features and distribution in oncology.
